# Supplementary material for: Structural and topological nature of plasticity in sheared granular materials
Source: Nat Commun. 2018 Jul 25;9:2911. doi: 10.1038/s41467-018-05329-8 (PMC6060108; doi:10.1038/s41467-018-05329-8)
Supplement: Supplementary file 1 — Supplementary Information [file 41467_2018_5329_MOESM1_ESM.pdf]

Supplementary Information

Structural and Topological Nature of Plasticity in Sheared Granular Materials

Yixin Cao et al.

Supplementary Table 1: List of sample initial conditions investigated

Sample	Γ(g)	W(d)	ϕ_0
1.5g_W=25d	1.5	25	0.636
1.5g_W=15d	1.5	15	0.629
4.5g_W=15d	4.5	15	0.623

Supplementary Table 2: Comparison between structural defects in crystalline and disordered granular packings

	**Crystalline solid**	**Disordered granular packings**
Structure order	crystalline order	glass order (regular tetrahedra)
Structure defect	dislocation	highly distorted tetrahedral<br>(4-ring disclination)
Plastic event	translational motion of dislocation	flip events<br>(rotation of 4-ring disclination)

Supplementary Note 1: Quasi-static plane shear

In the experiment, the shearing bracket (which is fixed to a motor) moves up with a fixed length for each shear step, which is shown by an almost linear relation between vertical displacement and shear step number n at $x = -10d$ in Supplementary Figure 1. The corresponding curve at the center of the shear band ($x = 1d$) shows a reduced but still almost constant slope. From the average particle vertical displacement $\langle \Delta r_{z,x=1d} \rangle$, we calculated the average strain for each shear step as $\Delta\gamma = 1.2 \pm 0.2\%$.

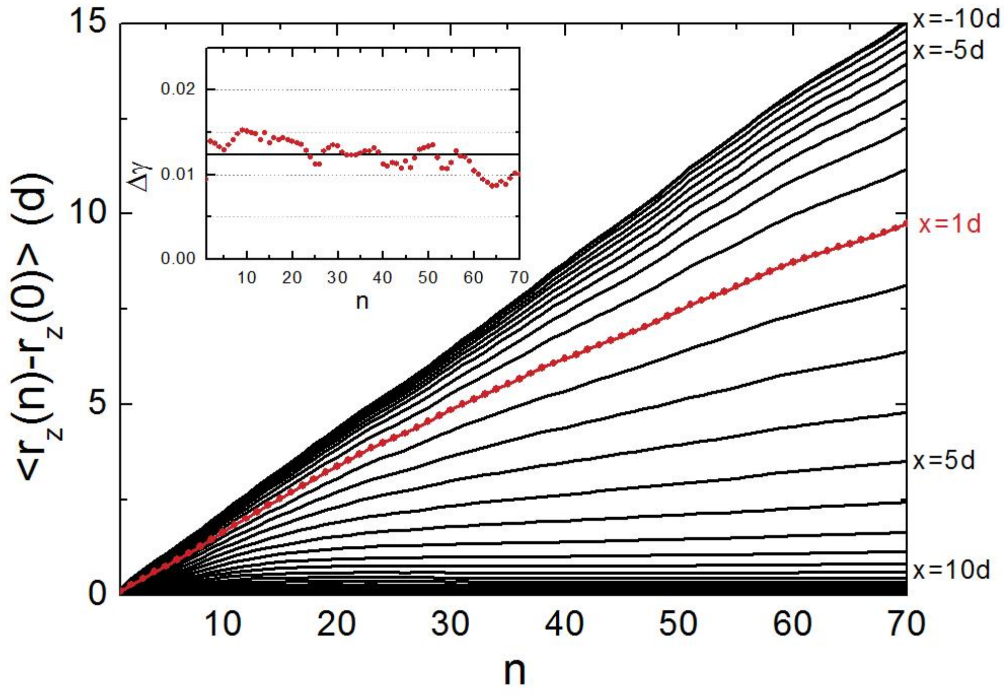

Supplementary Figure 1. Average vertical displacement versus shear step number *n*. The label $x = id(-10 \le i \le 26)$ denotes all particles within a slice centered at $x = id$ with a thickness of $1d$ along x direction. The average displacements of particles within different slices from their initial positions $r_z(0)$ after n shear steps are shown (the figure includes data only for sample 1.5g_W=25d, while all other samples show similar behaviors). The displacement in the center of shear band (x=1d) is highlighted in red. Inset shows the shear strain $\Delta\gamma$ for each shear step calculated from the average particle displacement $\langle \Delta r_{z,x=1d} \rangle$ at x=1d. The solid line denotes the average value of 1.2%.

Supplementary Note 2: Diffusion dynamics and Intermediate scattering function

Once critical state is reached, the shear band is considered to be in a continuous unbounded plastic flow state. Since local plastic deformations lead to structural rearrangements, we characterize particle diffusion dynamics in the shear band based on $\delta r_i (i = x, y, z)$. As shown in Supplementary Figure 2, it is found that initially the particles show a superdiffusive behavior and become diffusive after $\Delta\gamma = \gamma - \gamma_c \approx 20\%$. The diffusion curves along all three axes are quite similar which suggests the behavior is independent of the external shear direction.

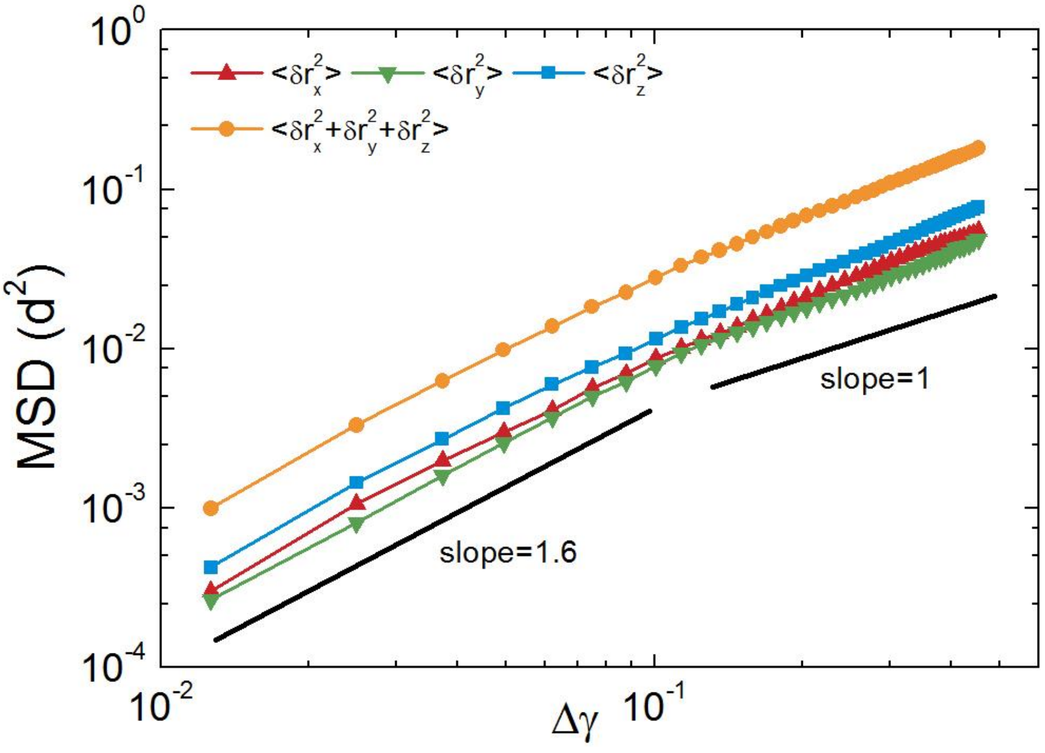

Supplementary Figure 2. The diffusion curves of particles inside the shear band after critical shear strain $\gamma_c = 40\%$.

In addition to diffusion curves, we also characterized the structural relaxation dynamics by the self-intermediate scattering function $F_s(k,\Delta\gamma)=\frac{1}{N}\sum_{j=1}^{N}\exp(ik\cdot\delta\vec{r}_j(\Delta\gamma))$, where j denotes any of the traced particles starting from the critical state γ_c to a state with a strain increment $\Delta\gamma=\gamma-\gamma_c$, and N is total number of particles. The curves in Supplementary Figure 3 is fitted with the following formula:

$$F_s(\Delta\gamma)=\mathrm{A}\exp[-(\Delta\gamma/\gamma^*)^\beta]+F_s(\Delta\gamma_{\max}).$$

The fitting parameters A=1.13, $\gamma^*=0.55$, $\beta=0.98$ and $F_s(\Delta\gamma_{\max})=-0.10$.

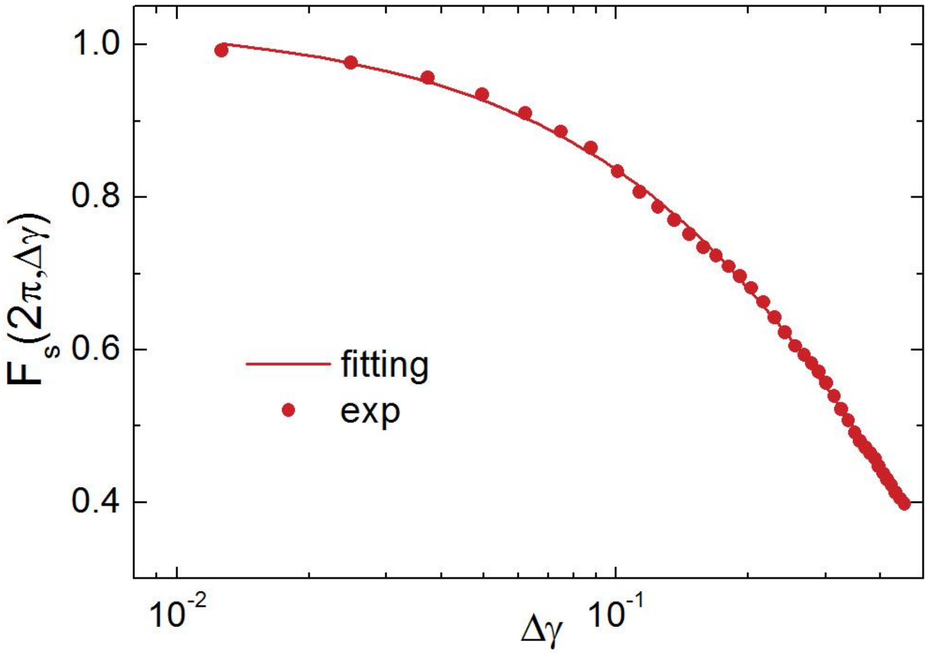

Supplementary Figure 3. Self-intermediate scattering function.

Supplementary Note 3: Nonaffine displacement

We define the nonaffine displacement of the particle i as $\delta r_i = \Delta r_i - \sum_{j=1}^{n} \Delta r_j$, where j denotes the n particles within a distance 2.5d from the particle i. As shown in Supplementary Figure 4, although the mean displacements after a shear step show non-negative values along both x and z axes, the corresponding mean nonaffine displacements are almost zero along all three axes, which suggests that the affine displacements have almost been removed using above method. We also compare the results obtained from this method with the scalar parameter $|D_{\min}|$ as introduced by Langer and coworkers[1]. They define

$$D^2(t,\Delta t) = \sum_n \sum_i \left(r_n^i(t) - r_0^i(t) - \sum_j (\delta_{ij} + \varepsilon_{ij}) \times [r_n^j(t-\Delta t) - r_0^j(t-\Delta t)] \right)^2,$$

where index i and j denote spatial coordinates and n run over the particles within a radial distance 2.5d from the central particle, $n = 0$ being the central particle. We then find $D_{\min}^2$ by calculating

$$X_{ij} = \sum_n [r_n^i(t) - r_0^i(t)] \times [r_n^j(t-\Delta t) - r_0^j(t-\Delta t)],$$

$$Y_{ij} = \sum_n [r_n^i(t-\Delta t) - r_0^i(t-\Delta t)] \times [r_n^j(t-\Delta t) - r_0^j(t-\Delta t)],$$

$$\varepsilon_{ij} = \sum_k X_{ik} Y_{jk}^{-1} - \delta_{ij}.$$

$|D_{\min}|$ is in quantitative agreement with $|\delta \mathbf{r}|$ other than a scaling factor as shown in Supplementary Figure 4.

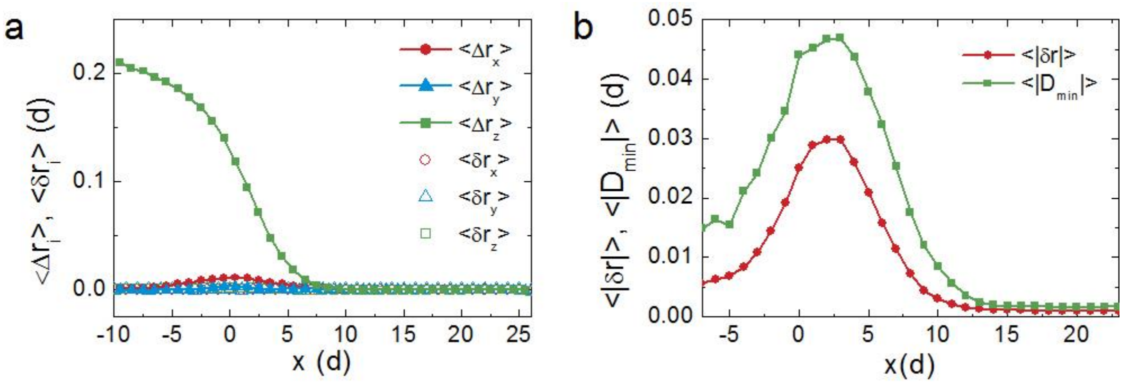

Supplementary Figure 4. Absolute and nonaffine displacement profiles along *x* direction

after a single shear step. a, absolute (solid) and nonaffine (open) displacements of x, y, and z directions over a single shear step are shown respectively. The average is taken over each $1d$-thick slice region centered at $x=id$, where i ranges from -10 to 26. **b**, The distributions of $|\delta\mathbf{r}|$ and $|D_{\min}|$ along x direction.

In Supplementary Figure 5, the nonaffine displacement profiles along x-direction for different shear strains are shown, in which strain localization can be clearly seen when shear band is formed.

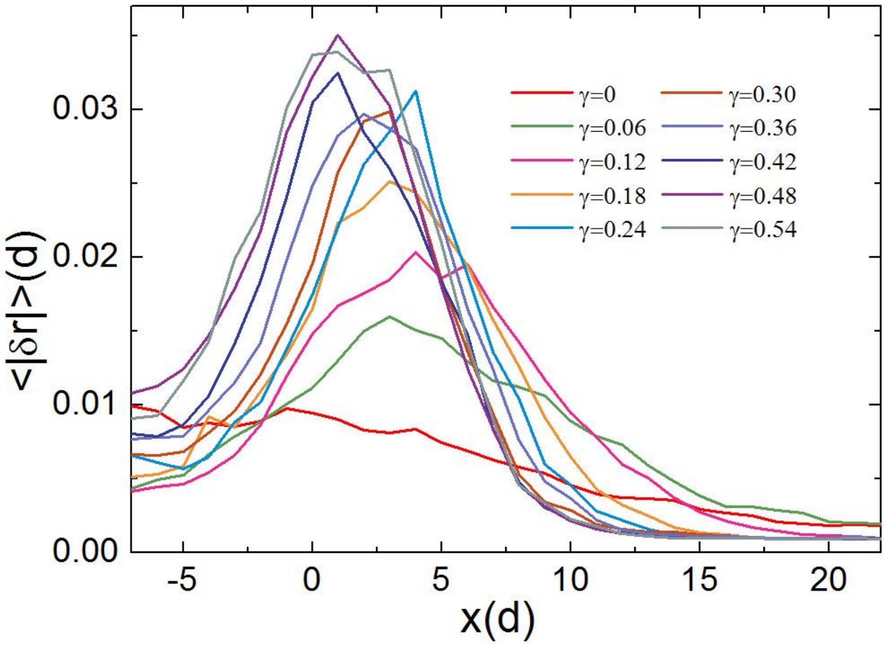

Supplementary Figure 5. Nonaffine displacement profiles along x-direction for different shear strains.

Supplementary Note 4: Evolution of tetrahedral shape parameter δ under shear

To understand how the shapes of tetrahedra evolve under shear, we track the evolution of the shapes of tetrahedra with different initial δ. It is evident that after sufficient shear strain, tetrahedra with different initial δ demonstrate similar shape distributions as shown in Supplementary Figure 6, which suggests that the system has reached steady state and the shape memory of a tetrahedron is finite.

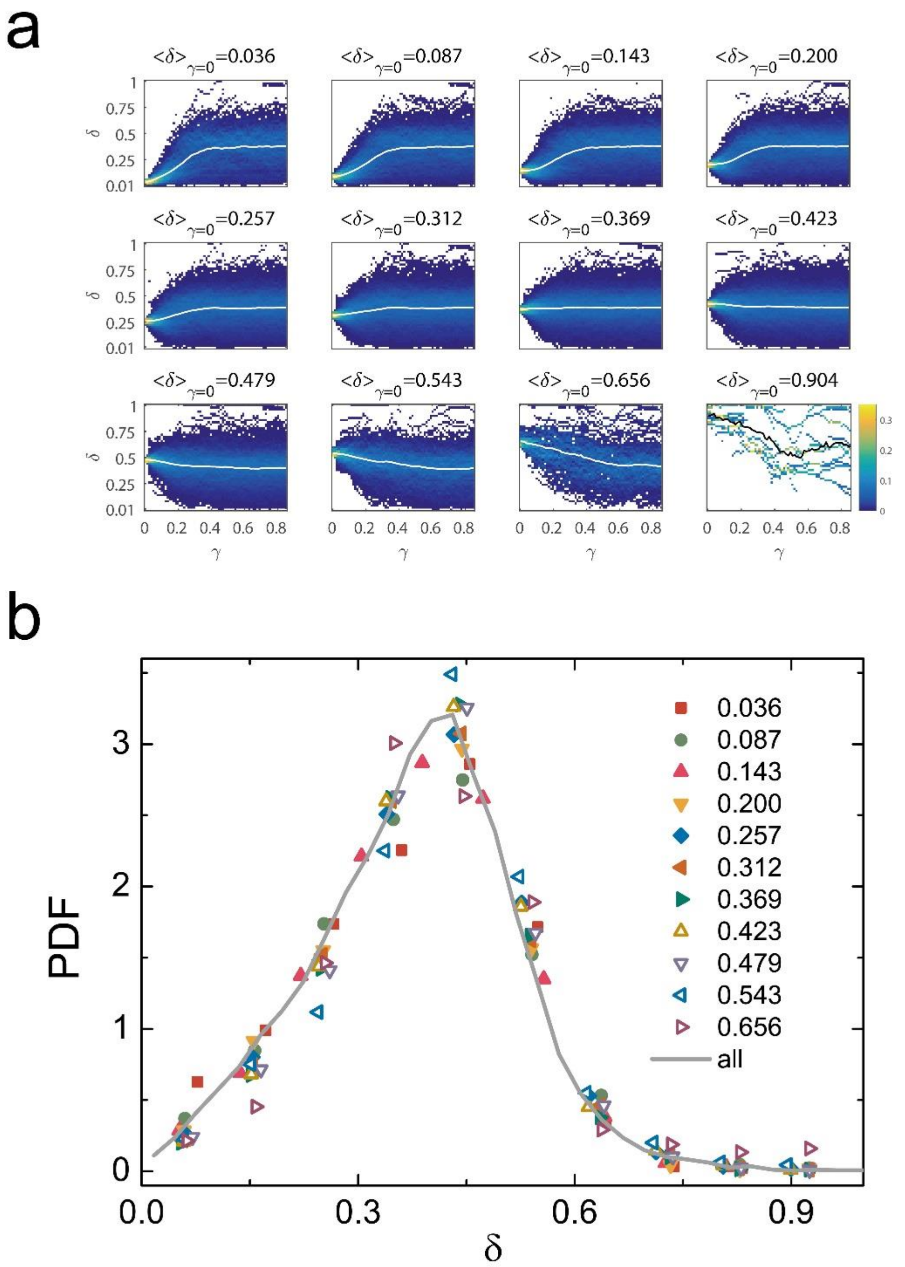

Supplementary Figure 6. Evolution of tetrahedra shape parameter δ under shear. a,

tetrahedra in the shear band are classified into 12 groups by their initial δ values at the beginning ($\gamma = 0$), and traced during the shearing process as γ increases from 0 to about 0.86. Each panel plots the evolution of δ distribution for each group (labeled by the initial average δ). The color map denotes the probability density $P(\delta)$. The solid lines show the evolutions of the average $<\delta>$ for each group with increasing γ. **b,** δ distributions for each group in **a** (symbols) and all tetrahedra (the gray line) at $\gamma = 0.86$.

Supplementary Note 5: Lifetime of tetrahedra under shear

Supplementary Figure 7 shows the probability $P_\delta(\Delta\gamma) = N_{maintain} / N_{tot}$ that tetrahedra with different initial δ maintain its four vertices, that is, not flip after a finite shear, where $\Delta\gamma = \gamma - \gamma_c$ and γ_c is the critical strain. It is obvious that the smaller δ is, the more stable the tetrahedron is. With a small shear step, the unstable tetrahedra are always bad tetrahedra (large δ). The good tetrahedra (small δ) have to first gradually transform into bad tetrahedra before becoming unstable.

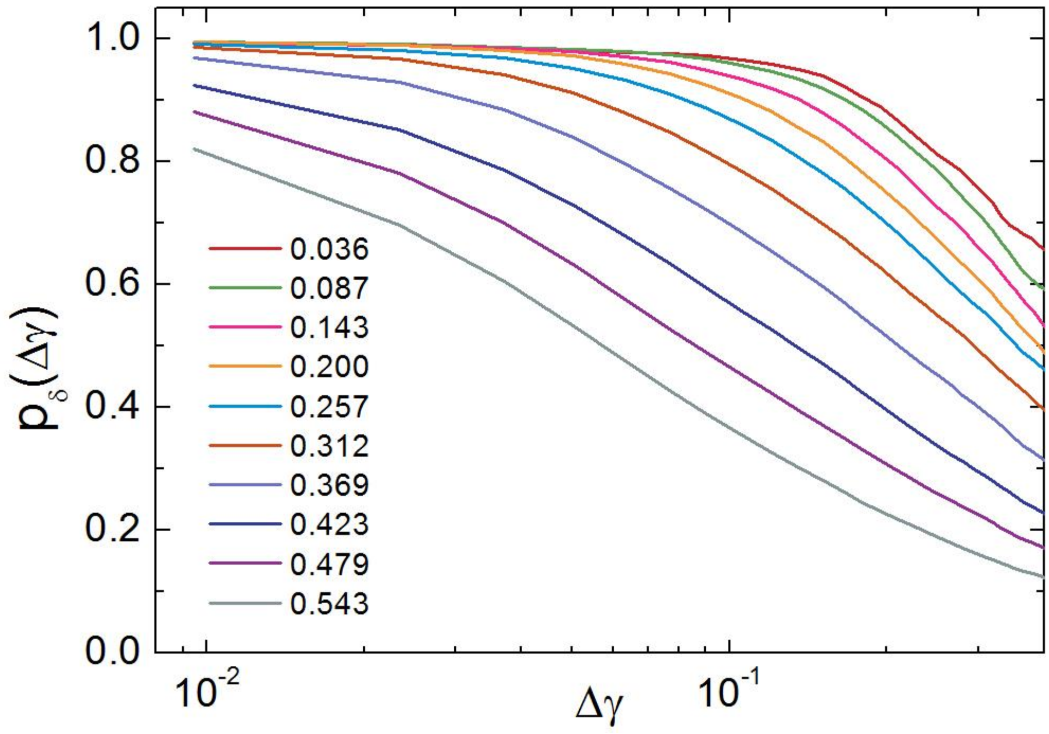

Supplementary Figure 7. Lifetime of tetrahedra of different initial δ upon shear. The probability of tetrahedra with different initial δ maintaining its four vertices after a finite shear strain**.**

Supplementary References

1 Falk, M. L. & Langer, J. S. Dynamics of viscoplastic deformation in amorphous solids. Phys. Rev. E 57, 7192-7205 (1998).
