# Supplementary material for: Structural and topological nature of plasticity in sheared granular materials
Source: Nat Commun. 2018 Jul 25;9:2911. doi: 10.1038/s41467-018-05329-8 (PMC6060108; doi:10.1038/s41467-018-05329-8)
Supplement: Supplementary file 3 — Description of Additional Supplementary Files [file 41467_2018_5329_MOESM3_ESM.pdf]

Description of Additional Supplementary Files

File Name: Supplementary Movie 1

Description: The movie shows the structural evolution of the particles in the imaging window during the shear process.
