# Supplementary figures and images for: Structural and topological nature of plasticity in sheared granular materials
Source: Nat Commun. 2018 Jul 25;9:2911. doi: 10.1038/s41467-018-05329-8 (PMC6060108; doi:10.1038/s41467-018-05329-8)

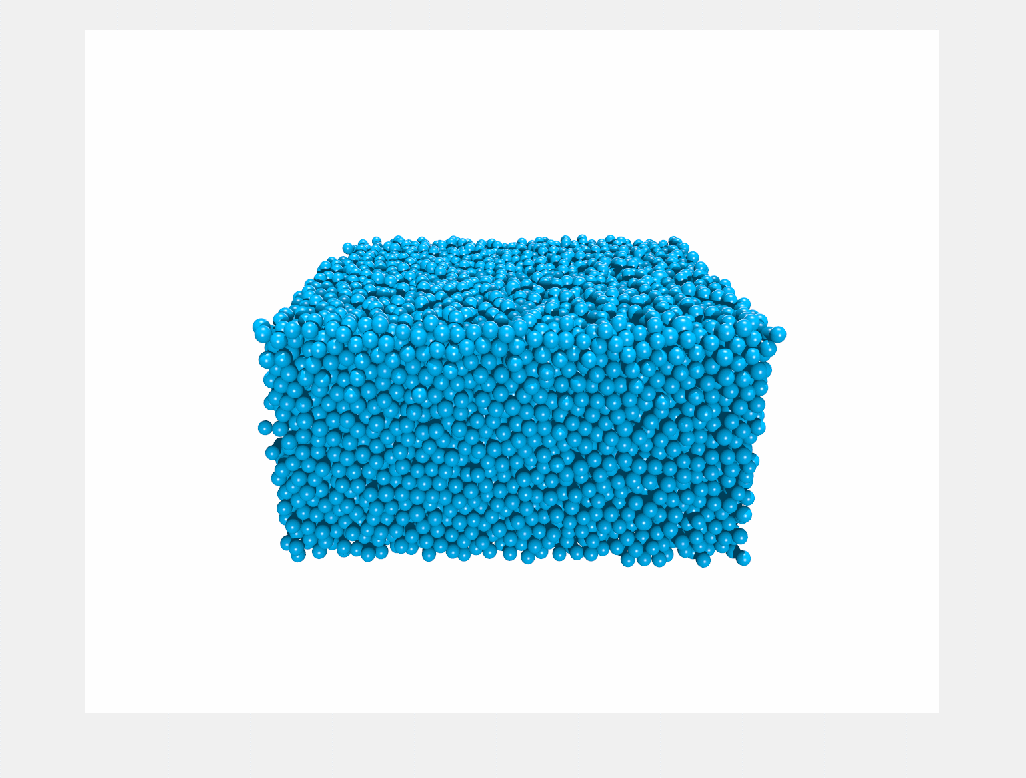

Supplement: Supplementary file 4 — Supplementary Movie 1 [file 41467_2018_5329_MOESM4_ESM.gif]
